# Challenges in developing capability measures for children and young people for use in the economic evaluation of health and care interventions

**DOI:** 10.1002/hec.4363

**Published:** 2021-05-25

**Authors:** Paul Mark Mitchell, Samantha Husbands, Sarah Byford, Philip Kinghorn, Cara Bailey, Tim J Peters, Joanna Coast

**Affiliations:** 1 Health Economics Bristol (HEB), Population Health Sciences, Bristol Medical School, University of Bristol, Bristol, UK; 2 King’s Health Economics (KHE), Health Service and Population Research, Institute of Psychiatry, Psychology & Neuroscience, King’s College London, London, UK; 3 Health Economics Unit (HEU), Institute of Applied Health Research, University of Birmingham, Birmingham, UK; 4 School of Nursing, Institute of Clinical Sciences, University of Birmingham, Birmingham, UK; 5 Population Health Sciences, Bristol Medical School, University of Bristol, Bristol, UK

**Keywords:** D63, I140, capability approach, outcomes research, economic evaluation, ICECAP measures

## Abstract

Methods for measuring outcomes suitable for economic evaluations of health and care interventions have primarily focused on adults. The validity of such methods for children and young people is questionable in areas including the outcome domains measured and how they are measured and valued, with most existing measures narrowly focusing on health. Novel methods for assessing benefits beyond health by focusing on a person’s capability have also concentrated on adults to date. This paper aims to set out the rationale for capability measures in children and young people. It argues for the need to expand the evaluative space beyond health functionings towards broader capabilities, with children and young people playing an integral role in capability measure development. Drawing from existing literature, specific challenges related to the identification, measurement, and valuation of capabilities in children and young people are also discussed. Finally, the practical implications for conducting economic evaluation when measuring and valuing capabilities at different stages across the life-course are illustrated. We develop an alternative framework based on conceiving capabilities as evolving across the life-course. This framework may also be helpful in thinking about how to model health outcomes across the life-course.

## 1 Introduction

The role of economic evaluation in the assessment of the value for money offered by health and care interventions has taken a prominent role in decision-making concerning the provision of health and care services (MacKillop & Sheard, 2018). Economic evaluation in health and care (DHSC, 2013) has developed from the traditional economic approach of cost-benefit analysis (where benefits and costs are both valued in monetary terms), to economic evaluation that primarily focuses on patient benefits in terms of health outcomes (Drummond, Sculpher, Claxton, Stoddart, & Torrance, 2015; Neumann, Sanders, Russell, Siegel, & Ganiats, 2017). In particular, the quality-adjusted life year (QALY), a combination of health status measurement and length of time in that health state (Weinstein, Torrance, & McGuire, 2009) has become the standard currency for economic assessments for new health technologies and clinical guidelines for regulatory bodies such as the National Institute for Health and Care Excellence (NICE) in England (NICE, 2014) and comparable bodies internationally (Rowen, Azzabi Zouraq, Chevrou-Severac, & van Hout, 2017). The EQ-5D is internationally the most widely used patient-reported outcome measure (PROM) to generate the health states for QALY calculations in health technology assessments (Wisløff et al., 2014), though others are also used (Richardson, Iezzi, Khan, Chen, & Maxwell, 2016).

Although analytic approaches have been tailored for use in health and care interventions, there remain problems in applying the recommended economic evaluation methods for generating QALYs across all population groups. For example, difficulties in applying standard economic approaches to patients near the end-of-life have been discussed (Coast, 2014; Normand, 2009; Round, 2012, 2016). Another important population group where challenges in applying the standard approach are evident, is children and young people (CYP), defined here as those aged under 18 years old (Detrick, 1999). Methods for conducting economic evaluation have ostensibly been developed to cover all members of the general population, but both methodological development and practical application have focused primarily on the adult population, with methods used for adult evaluations regularly transposed to CYP evaluations. This is potentially problematic given the differences between adult and CYP population groups in terms of development, understanding and age-specific behaviours. A consultation in the Netherlands (Dirksen & Evers, 2016) identified numerous methodological challenges when applying standard economic evaluation methods in CYP. The most prominent issues related to outcome identification, outcome measurement and outcome valuation (as well as cost valuation and time horizon/analytic approach) (Dirksen & Evers, 2016).

A review of the valuation of health states for QALYs in CYP found that the EQ-5D adult version was most frequently used (Thorrington & Eames, 2015). This suggests a shift in practice towards greater compliance with the recommendations of regulatory bodies to standardise health economic outcomes. This shift contrasts with previous review findings (Griebsch, Coast, & Brown, 2005), and is perhaps surprising given the rise of condition-specific (Solans et al., 2008) and generic health (Chen & Ratcliffe, 2015) measures available for CYP. It is also unexpected, given the growing recognition that a “one-size-fits-all” approach may be inappropriate when conducting economic evaluations across health and care, with alternatives to QALYs being allowed by NICE in public health and social care (NICE, 2014). Adopting a broader perspective may allow for comparisons of interventions across the public sector that try and improve CYP outcomes not only in health care, but also in social care, justice and education (Noyes & Edwards, 2011).

An alternative economic evaluative paradigm that shifts attention to individual capabilities is one option that has been taken up by decision-makers in England for social care (NICE, 2014) and in the Netherlands for long-term conditions (ZorginstituutNederland, 2016). A focus on capabilities – that is, whether a person *is able* to do and be the things in life that are of value to them (Sen, 1987, 1993), is argued to offer a richer evaluative space than the current approach which (i) limits the focus to specific health functionings, and (ii) focuses only on what a person actually does, without consideration of whether they are able to do it, even if they may choose not to do so (P. Anand & Dolan, 2005; Coast, Smith, & Lorgelly, 2008a; Verkerk, Busschbach, & Karssing, 2001). Capability measures for use in economic evaluations of health and care interventions, including public health, chronic pain and mental health, have only focused on adults to date (Al-Janabi, Flynn, & Coast, 2012; Coast, Flynn, et al., 2008; Greco, Skordis-Worrall, Mkandawire, & Mills, 2015; Kinghorn, Robinson, & Smith, 2015; Lorgelly, Lorimer, Fenwick, Briggs, & Anand, 2015; Simon et al., 2013; Sutton & Coast, 2014).

The aim of this paper is to discuss the challenges of developing capability measures for CYP to use in economic evaluation of health and care interventions. The remainder of the paper is structured as follows. Section 2 provides a summary of the existing literature on outcomes for CYP used in health economics currently, drawing from several existing reviews in this area. Section 3 shifts focus towards the capability approach and the rationale for measuring individual capabilities. Section 4 looks at the applicability of the capability approach to CYP. Section 5 addresses specific challenges with developing CYP measures. Section 6 outlines a framework that allows for different measures across the life-course. Section 7 presents conclusions from the paper.

## 2 Children and Young People Outcomes in Health Economic Evaluation

Several detailed reviews have been conducted on challenges specifically related to the measurement and valuation of health outcomes in CYP. One review details the generic health measures that have been developed or adapted from adult measures for use in children and young people (Chen & Ratcliffe, 2015). For challenges relating to measuring health outcomes in CYP, other reviews have focused on questions over what is the appropriate health outcome measure to use, with many economic evaluations continuing to rely on the use of measures developed for adults to generate adult health states and the application of adult values (Hill, Rowen, Pennington, Wong, & Wailoo, 2020; Kwon et al., 2019).

There are also numerous questions with regard to the valuation of health states in CYP populations, including the perspective to take when valuing CYP health states (Rowen, Rivero-Arias, Devlin, & Ratcliffe, 2020). Evidence suggests that adult and CYP values differ (Kind, Klose, Gusi, Olivares, & Greiner, 2015; Ratcliffe et al., 2016).Parental valuation of CYP health states have been shown to overestimate values (Kwon et al., 2018). Even when adults have valued both adult and youth versions of the same measure (i.e. the adult and youth versions of the EQ-5D), different values have been generated for each measure version (Kreimeier et al., 2018), thereby raising questions over the applicability of adult health state values applied in economic evaluations for CYP populations.

There are also more practical questions concerning the conduct of an economic evaluation if drawing on multiple sources for measuring and valuing health states at different stages in life (Hill, Rowen, Pennington, Wong, & Wailoo, 2019). Although there are likely to be overlapping challenges in developing CYP specific measures, the remainder of this paper will attempt to address challenges specifically regarding the development of capability measures in CYP.

## 3 The Capability Approach

Initially developed by Amartya Sen (Sen, 1987, 1992, 1993) and Martha Nussbaum (Nussbaum, 2001, 2011), the capability approach has become a popular normative framework to use across many disciplines and sectors of society (Robeyns, 2017). These include a diversity of applications in health and care sectors, such as research on disability policy (P. Anand, Roope, Culyer, & Smith, 2020; Burchardt, 2004; Kuklys, 2005; Mitra, 2006), global health ethics (Venkatapuram, 2011) and universal healthcare coverage (Ruger, 2010). There has also been a large amount of research concerning the application of the capability approach to the development of broader measures of patient benefits in health and care economic evaluations (Helter, Coast, Łaszewska, Stamm, & Simon, 2020).

At the core of Sen’s capability approach is an argument to shift evaluative focus away from utility and consumption-based estimates of how well-off people are in society. Instead, he argues for the evaluative focus to shift towards what capabilities (also referred to as opportunities, freedoms, advantages) people have to pursue the functionings, the beings and doings, that are valuable to them (Sen, 1993). To illustrate the difference in functionings and capabilities, Sen argues that focusing on functionings alone may obscure analysis from important differences between people. For instance, a person fasting and one starving may achieve the same levels of undernourishment, but by knowing about their differing capabilities to be nourished, it leads us to a greater insight into the well-being of individuals, which would otherwise be excluded from our analysis (Sen, 1992).

Sen’s critique of welfare economics has been used (Brouwer, Culyer, van Exel, & Rutten, 2008), albeit mainly post hoc (Coast, Smith, & Lorgelly, 2008b) by health economists to justify the shift towards health functioning measures such as quality-adjusted life years (QALYs) (Culyer, 1989) and disability-adjusted life years (DALYs) (Murray & Acharya, 1997). While some health economists feel that Sen’s capability approach can be captured using existing QALY approaches (Bleichrodt & Quiggin, 2013; Cookson, 2005), others disagree. The key argument made for a shift from QALY and DALY metrics in this area is the argument that there is a need to expand evaluative focus beyond health outcomes, to also include non-health outcomes (Brazier & Tsuchiya, 2015), with some arguing for the need to expand the evaluative space from people’s functionings to their capabilities to fully operationalise the capability approach (Coast, Smith, et al., 2008a). If analysis relies on functionings alone, there is an argument as to whether any additional insights can be gained in applying the capability approach in practice (Robeyns, 2006).

## 4 The Capability Approach Applied to Children and Young People

As initially developed, the capability approach, like other liberal theories of justice, assumes an individual’s ability to act rationally and make decisions over their functioning possibilities (or ‘capability set’). This approach largely inhibits the inclusion of those who do not or are not yet perceived to have fully developed the capacities to undertake such decisions, including CYP. Sen and Nussbaum, the initial capability approach architects, have paid little attention directly to CYP well-being. When discussed, what is important is for CYP to develop into adults who are able to make decisions about what functionings they want to pursue in adulthood, with little attention on how to asses current levels of well-being for CYP (Comim, Ballet, Biggeri, & Iervese, 2011). It is notable, however, that the Human Development Index developed in part by Sen for the United Nations human development reports (S. Anand & Sen, 1994) consists of functionings associated with childhood in terms of health (life expectancy) and basic educational attainment (Comim et al., 2011).

In recent years, researchers have advanced the capability approach to encompass CYP. Ballet et al. (2011), for example, argue for a framework based on “evolving capabilities”, with a recognition that CYP have autonomy or agency and this is likely to develop as they age and mature (Ballet, Biggeri, & Comim, 2011). Such a framework places emphasis on the role and voice of CYP, whilst also recognising that capability formation in CYP is largely influenced by their parents/guardians and provisions of the state (Ballet et al., 2011). Others have focused on evaluating educational policy and have argued that a central focus should be on the CYP’s capability to aspire (Hart, 2014b). The key emphasis for most research concerning the capability approach and CYP has been to highlight a role for CYP in identifying, measuring and valuing their own well-being (Biggeri, Ballet, & Comim, 2011; Hart, Biggeri, & Babic, 2014; Leßmann, Otto, & Ziegler, 2011).

## 5 Challenges in Applying the Capability Approach to Children and Young People

### 5.1 Capabilities versus functionings

Although there is a compelling argument for the need to go beyond health related functionings to broader capabilities (Coast, Smith, et al., 2008a), whether this also holds true for CYP as it does for adults may require some further justification. Neither Sen nor Nussbaum has dedicated much analysis towards implementing a capability approach for CYP, and there has been some question over its practical application in this population (Saito, 2003). One ongoing debate in the capability approach is over whether there is a need to go beyond the assessment of functionings (Robeyns, 2017). There is an argument that CYP capabilities can only be assessed later, following the development of important functionings in their formative years (Schweiger & Graf, 2015).

Another argument has been made that, as capabilities are currently unobservable in routinely collected data, there is a requirement to only focus on observable functionings (Krishnakumar, 2007) and most empirical CYP research within the capabilities approach has drawn from functioning data either as proxies for capabilities (P. Anand & Roope, 2016; Domínguez-Serrano & del Moral Espín, 2018; Phipps, 1999) or to estimate capabilities as latent variables in structural equation modelling (Addabbo & Di Tommaso, 2011; Volkert & Wüst, 2011).^1^

Although the argument for choosing functionings over capabilities as the well-being evaluative space is appealing when it comes to analysing existing data sources, this argument appears weaker for primary data collection, as is common practice in economic evaluations for trial-based health and care interventions. Indeed, within the education literature on the capability approach, it has been noted that focusing just on core educational functionings such as reading, writing and arithmetic may not provide an adequate evaluative space to understand the development of CYP’s autonomy, agency and aspiration (Ballet et al., 2011; Hart, 2014b). Capabilities such as ‘the capability to aspire’ have been argued to be a ‘meta-capability’ in that they are instrumental for enabling future opportunities to flourish (Hart, 2014b).

Some of the potential concerns about attempting to directly measure capabilities have also been addressed in the adult context: research has shown that the measurement of capability well-being or “perceived capabilities” (Van Ootegem & Verhofstadt, 2015) is feasible (Al-Janabi, Flynn, Peters, Bryan, & Coast, 2015; Al-Janabi, Keeley, Mitchell, & Coast, 2013); and concerns that direct measurement of capabilities might result in ‘adaptive preferences’ (whereby individuals may adjust their expectations of what is possible based on a reference group) that may obscure inherent deprivations between groups as with utility measurement (Sen, 2002; Teschl & Comim, 2005) were not evident for capability well-being measures at the end of life for adults (Coast et al., 2018). Whether these issues are more acute for CYP remains to be determined.

A focus purely on health functionings, important as they are for CYP development, may exclude other important information. Concentrating only on health may not adequately capture the broader impacts on CYP’s well-being from poor health, such as lost education and play time with friends. A focus only on functionings does not enable the concerns raised by Hart around issues of autonomy, agency and aspiration to be addressed (Hart, 2016). Therefore, the need for broadening the evaluative space in health economics from health functionings to a broader conception of capabilities appears as strong as the argument for making this shift in evaluations for adults, and the conceptualisation of capabilities may even need to be broader.

The capability to aspire, noted as important for CYP, can also be linked with a question about whether capabilities for CYP relate just to current well-being or should focus also on future well-becoming, that is, the opportunities for development a CYP has. The emphasis on measuring benefits to CYP has generally, until recently, been on well-becoming and is changing to encompass both perspectives (Ben-Arieh, 2008). By contrast, in health economics insofar as health benefits to CYP have been distinguished from those of adults, they have largely concentrated on well-being rather than well-becoming (Chen & Ratcliffe, 2015). It may be important, in taking forward a capability approach to CYP, to focus on “opportunities for present and future functioning” ((Biggeri & Santi, 2012), p.375) – that is, on well-being and well-becoming – as both are likely to form important components of value during these stages of life that are crucial for development (Heckman, 2006). Better understanding of the importance of well-becoming to CYP can potentially be explored using qualitative research methods (Coast, 2017).

### 5.2 Participation versus paternalism/expert-led approaches

A second key debate within the capability approach generally is the role of participatory methods in identifying capabilities. There are those who favour an expert led approach (Robeyns, 2003), a number of whom have used Nussbaum’s universal list of central human capabilities (Nussbaum, 2011) as a helpful starting point for identifying relevant data. Others favour a combination of drawing from an existing list of capabilities, but using this alongside participatory methods, and this approach has been used in developing a list of capabilities for children (Biggeri, Libanora, Mariani, & Menchini, 2006). The capability list developed by Biggeri and colleagues (2006) (see Table 1), was developed using methods similar to approaches taken for the OxCap suite of adult capability measures developed for mental health (Simon et al., 2013) and public health (Lorgelly et al., 2015). Mixed approaches of these types are often used in developing health measures in health economics, where domains initially identified are typically expert led, with patient and public participation usually limited to the measurement and valuation stages of generating QALYs (Pickles et al., 2019).

Reliance on experts is a challenge to liberal theories of justice, including welfare economics, as it moves away from the individual as the key arbiter of what is best for them (Brouwer et al., 2008). Sen too, has favoured a participatory approach to identifying capabilities (Sen, 2004) and one clear departure from a more paternalistic approach to measure development has been the development of the ICECAP capability well-being measures (Al-Janabi et al., 2012; Coast, Flynn, et al., 2008; Sutton & Coast, 2014). Since the first ICECAP capability measure was developed for older people (Coast, Flynn, et al., 2008; Grewal et al., 2006), there has been an emphasis on the population of interest identifying for themselves the areas of life that are most important to them: for the general adult population (Al-Janabi et al., 2012) and adults near the end of life (Sutton & Coast, 2014) as well. This broader participatory approach more clearly diverges from expert-led approaches and resonates with those adopting a greater role for individual participation in the capability approach.

There is already evidence from health economics, through the development of the Child Health Utility 9 Dimension (CHU-9D), that a participatory approach involving CYP themselves is feasible for identifying items for CYP (Katherine Stevens, 2010). Combined with evidence from Biggeri et al. (2006) that CYP can discuss capabilities, it seems that a participatory approach to developing CYP measures is both feasible and fits within liberal theories of justice (Ballet et al., 2011). However, a balance is likely to be required to account for current well-being and future well-becoming, where these perspectives can be seen as obtaining complementary information (Gardner, 2015).

### 5.3 Identification of capabilities for children and young people

The identification of capabilities for CYP offers some additional challenges when compared to the identification of capabilities for adults. For adult ICECAP measures, for example, adults were interviewed to identify what mattered to them in their lives in order to determine the capabilities to include in the measure (Al-Janabi et al., 2012; Grewal et al., 2006; Sutton & Coast, 2014). For some stages of development of CYP, of course, it will be necessary to gather the views of others, such as their primary caregivers (e.g. parents/guardians), as infants and very young children, as well as some older CYP lacking capacity, will not be able to identify relevant and important capabilities themselves. The views of others may also be important in understanding issues around particular capabilities around aspiration and future well-becoming. One could argue there is a role for many different stakeholders in CYP development, from family members, to professionals in education and health and care, who are likely to bring different perspectives on what is considered important for CYP.

Nevertheless, it is important not to underestimate CYPs’ abilities to understand concepts that some adults may think are beyond their abilities, with evidence showing that young children are able to understand health and illness concepts (Bevans & Forrest, 2010). Research studies are increasingly seeking ways to facilitate the involvement of CYP in research (Clark & Statham, 2005), including through the use of creative, innovative or participatory (CIP) research methods to encourage an environment where children can, and do, feel comfortable in putting forward their opinions and experiences (Davis, 1998; Kirk, 2007). The use of such CIP methods is based on the view that traditional qualitative approaches, for example interviews, are less appropriate for use with children, particularly those who may not be as able to articulate their opinions using only formal or language-based methods (Punch, 2002; Shaw, Brady, & Davey, 2011; Whale, 2017).

A potential strategy for generating information about important capabilities from CYP is to use a “draw, write and tell” methods approach, where the child or young person is encouraged to produce artwork and/or short written excerpts around the research topic and explain to the researcher what they have produced (Angell & Angell, 2013). The advantage of drawing is that it is a relatively quick, simple and efficient method of data collection (Fargas-Malet, McSherry, Larkin, & Robinson, 2010), and can act as a trigger for CYP to begin to discuss relevant issues, particularly personal topics (Arbuckle & Abetz-Webb, 2013; Fargas-Malet et al., 2010; Whale, 2017). The method can be adapted to the age and preferences of individual CYP, with them being given the option to draw, provide short written summaries or combine the two. Draw and write approaches have been applied successfully in child health research previously (Bevans & Forrest, 2010). This suggests that the method could be used to encourage CYP to think about and report their capabilities, by asking them to draw the things that are important to them now, but also what they want in the future. This would then allow the researcher the opportunity to ask the child or young person questions about their drawings and annotations, with the questions aimed at identifying the reasons why these things are important to them, to determine the overarching capabilities that should be included in the measures. Although some suggest that CIP methods can potentially distract from research aims (Arbuckle & Abetz-Webb, 2013) and produce research data that cannot be analysed, drawing methods used alongside qualitative interviews or questioning can provide a way to comfortably engage children and young people in research, whilst also producing meaningful qualitative data that can be analysed to identify relevant capabilities for different groups of CYP.

It should also be noted that, as with other stages of life, what capabilities matter to CYP and how much they matter is very likely to vary by cultural context. Although capability measures for adults are being used internationally (Afentou & Kinghorn, 2020; Proud, McLoughlin, & Kinghorn, 2019), cultural context is vital and adjustments to participatory approaches may also be needed for different cultural settings to ensure that capabilities being captured for CYP are appropriate and relevant (Greco, Lorgelly, & Yamabhai, 2016).

### 5.4 Measurement of capabilities in children and young people

A key challenge of developing measures for CYP is deciding how best to measure their capabilities, given that their quickly evolving development means that CYP are a heterogeneous group with differing needs linked to particular stages of development (Matza et al., 2013; Ungar & Gerber, 2010). Development stage influences not just what attributes matter most, but also the ability to self-complete a measure.

One option would be to follow guidance from Ungar and Gerber (Ungar & Gerber, 2010) by developing measures related to childhood dependency by age, whereby CYP reporting before the age of 5 is unlikely, ages 5-7 requiring pictograms to aid completion with parental help, ages 8-12 with modified language, and 12 years upwards allowing for an independent child report (Ungar & Gerber, 2010). This separation by these four age groups has been recommended as a starting point by guidelines for developing measures in paediatric populations, but development stage of the CYP should also be considered (Matza et al., 2013). It might be that age is not the most relevant factor upon which to base the choice of measure. Instead it might be more appropriate to focus measures on stages of child development related to cognitive ability and/or autonomy.

If capability measures are to be self-completed by CYP, there may be important aspects of measure development related to holding CYP’s attention span whilst minimising reliance on vocabulary to complete measures. Where CYP are deemed unable to assess their own state, parental proxy is the most commonly used method. When testing inter-rater reliability between parents and CYP who are able to self-report, agreement is stronger in observable dimensions (Bevans & Forrest, 2010). A common approach when measuring health-related quality of life is that parents complete on behalf of CYP who are unable to do so (Eiser & Morse, 2001). Health and care professionals are also sometimes used as proxies in health measures for generating QALYs for CYP (Kwon et al., 2019). When it comes to measuring capabilities for CYP there may be other stakeholders who could arguably give a more “objective” perspective, such as teachers or health and care professionals. Such challenges are not limited to CYP and similar measurement challenges occur in later life too (Coast, 2014).

### 5.5 Valuation of capabilities in children and young people

There have been a number of limitations with standard health economics methods for valuing child health states. Direct utility assessment methods such as time-trade off and standard gamble, commonly used for eliciting health state preferences from adults, are conceptually difficult tasks, with problems reported in using such methods with CYP (Ratcliffe et al., 2011; Saigal et al., 1998). Asking CYP to trade-off health with time is also likely to be too cognitively demanding for most CYP (Bevans & Forrest, 2010), as well as ethically challenging (Ratcliffe et al., 2011). Willingness to pay methods drawn from welfare economics also have limitations, as they require CYP to have a good understanding of the value of money and meaningful amounts to trade, both of which are unlikely for most CYP, who do not generally have direct control of their finances. Willingness to pay methods have also been shown to suffer from extreme altruism in valuation tasks conducted by parents and also by community samples when it comes to valuing child health (Bevans & Forrest, 2010; Prosser et al., 2004).

An alternative would be to employ valuation tasks using best-worst scaling (BWS), that have been shown to perform better in adolescents when compared to other valuation tasks (Ratcliffe et al., 2011). BWS profile case (Case 2), where “best” and “worst” choices are made on attribute levels presented as profiles (Louviere, Flynn, & Marley, 2015) is the method used for valuing previous ICECAP measures (Coast, Flynn, et al., 2008; Flynn et al., 2015; Huynh, Coast, Rose, Kinghorn, & Flynn, 2017). Although some researchers argue for no role of preferences to be compliant with the capability approach normative framework (Robeyns, 2017), the key argument for using BWS within the capability approach is that the random utility theoretical framework is less reliant on transitive preferences (Dagsvik, 2013). BWS has been shown to be feasible to complete for CYP as young as 10 years of age (Katherine Stevens, 2015), with more recent evidence from Australia and Spain suggesting adolescents, aged 11-17 years old, produced values for EQ-5D-Y that are valid when compared to an adult sample (Dalziel et al., 2020).

Valuations could therefore feasibly be obtained from older CYP, a parent sample, adult members of the general population or some combination of these three groups. One particular concern with relying on parent values is they tend to produce results inconsistent with those from CYP (Khadka, Kwon, Petrou, Lancsar, & Ratcliffe, 2019; Kwon et al., 2018). Public deliberation is another alternative for valuing capabilities in a way that may be more consistent with the capability approach in populations who may not be able to value for themselves (Kinghorn, 2019). Alternative methods for valuation have been proposed elsewhere in the capability literature too, using more normative and data-driven methods to elicit values (Greco, 2018).

Even if CYP cannot be fully involved in the valuation exercise, there may be ways to get their views on who they think should be involved in such decisions, using methods such as ‘hierarchical mapping’, whereby pictorial tools are used to explore network composition (Canaway, Al-Janabi, Kinghorn, Bailey, & Coast, 2019). Drawing from multiple viewpoints may be theoretically attractive when it comes to identifying, measuring and valuing CYP capabilities as it expands beyond a single subjective perception. It would, however, present practical issues associated with how to aggregate across these multiple viewpoints.

## 6 Icecap, Children and Young People and the Life-Course

The ICECAP suite of capability measures has shifted the focus of health economic outcome measures towards the stage of life that people find themselves in, whether that is life after the typical age of retirement as captured by the ICECAP for Older adults (ICECAP-O) (Coast, Flynn, et al., 2008; Grewal et al., 2006), or adults reaching the end of their life as captured through the ICECAP Supportive Care Measure (ICECAP-SCM) (Coast, 2014; Sutton & Coast, 2014). Drawing on this work, it has recently been proposed that evaluation should shift towards a ‘life-course’ approach that explicitly accepts differing values at different stages of life. The notion of the life-course focuses on the interlinking between generation and age, with influences on development being the historical context, social norms and generational influences into which a person is born, as well as their age (Elder, 1994). These factors, also relevant to the development of personal values (what is valued and what there is reason to value), depend on age, but also on the context in which that age is experienced. For example, we might expect that, a priori, population values have shifted due to the COVID-19 pandemic. A life-course approach is also helpful in thinking about CYP transitioning from different life stages, such as education to occupation or from adolescence to adulthood (Grundmann, Steinhoff, & Edelstein, 2011), as well as identifying specific points across the life-course where public policy intervention might be most beneficial (Yaqub, 2008).

To implement a life-course framework in practice requires decisions on how to shift between different measures along the life-course. For instance, age could be a way to decide what measure to use at different ages. This has been the recommendation used for the development of patient reported outcome measures by an expert taskforce on the issue (Matza et al., 2013). However, when it comes to capabilities, it seems that age might not necessarily be the appropriate criterion, as age might not adequately reflect differences between individuals, such as those who have special educational needs (Devecchi, Rose, & Shevlin, 2014).

Given that the development of CYPs’ agency and autonomy are seen as instrumental in developing future capabilities (Hart, 2014b), this might be a more relevant factor. However, this approach would encompass further difficulties in requiring a measure of agency/autonomy to judge what capability measure might be appropriate. Another approach that appears to fit within both a life-course approach and that of the capability approach is to assign measures based on the stages of transition, such as how different educational stages might emphasise getting CYP ready for the next education stage (Hart, 2014a). Choices that CYP make when transitioning towards adulthood are likely to play a part in future capabilities that are available to them (Bartelheimer, Büttner, & Schmidt, 2011; Cunha, Heckman, & Schennach, 2010).

In table 2, two existing approaches for identifying different groups of CYP are illustrated. There are various perspectives as to how child growth and development should be measured from a capability perspective (Sadlowski, 2011; Yousefzadeh, Biggeri, Arciprete, & Haisma, 2019). How many CYP capability measures that are eventually required will be largely driven by how different capabilities are between CYP at different stages of development as identified in the empirical research. This could, for example, focus on CYP measures based on age grouping (Andresen & Fegter, 2011; Babic, Graf, & Germes Castro, 2010) or distinguishable levels of autonomy at different stages of development. What is likely to be important across CYP capability measures is a consideration of both current well-being and future well-becoming in a manner that is consistent with the capability approach (Schweiger & Graf, 2015), as well as the differing levels of autonomy across CYP as they develop.

Of course, developing such a life-course approach, for CYP and beyond, to capability estimation will also require further thinking around the process of generating decision rules for use in economic evaluation. Although progress has been made on this for the capability approach (Kinghorn, 2019; Mitchell, Roberts, Barton, & Coast, 2015), the use of shifting measures across the life-course needs further consideration. This challenge also applies across health economic evaluations unless a single measure of health is used across the life-course.

## 7 Conclusion

This paper has outlined the rationale for adopting an evaluative focus on capability when it comes to health economic evaluation for interventions concerning CYP. We make the argument for embracing an evolving capabilities conceptual framework (Ballet et al., 2011), that takes into consideration CYP capabilities, whilst also recognising that their role in identifying, measuring and valuing those capabilities is likely to increase as the autonomy of CYP develops. This approach also allows for the development of an economic evaluation framework that places different capabilities at the centre of attention depending on where an individual is at in their life-course trajectory, and incorporates issues around developing the capability to aspire (Hart, 2016) by being open to capturing both capabilities for current well-being and future well-becoming.

Although the participation of younger children and those with limited capacity for other reasons (Devecchi et al., 2014) raises additional methodological questions, an approach focusing primarily on the perspective of the CYP’s capability well-being and well-becoming is consistent with the participatory-led approach adopted in the development of the ICECAP adult capability well-being measures, whilst allowing for questions of aspiration. Using similar participatory methods is consistent in developing a life-course framework where individuals are able to express what capability matters to them and how much it matters (Coast, 2019). As other health economists are also grappling with the methodological challenges in using child health measures alongside adult measures (Hill et al., 2019), the life-course framework we have outlined for capabilities may also offer some insights for other types of CYP health economic evaluations.

## Figures and Tables

**Table 1:** List of children and young people (CYP) capabilities (from Biggeri et al. 2006)

Capability	Meaning
1. Life and physical health	Being able to be physically healthy Enjoy a normal length of life
2. Love and care^†^	Being able to love and be loved by those who care for us Being able to be protected
3. Mental well-being	Being able to be mentally healthy
4. Bodily integrity and safety	Being able to be protected from violence of any sort
5. Social relations^†^	Being able to enjoy social networks and to give and receive social support
6. Participation^†^	Being able to participate in public and social life and to have a fair share of influence Being able to receive objective information
7. Education	Being able to be educated
8. Freedom from economic and non-economic exploitation	Being able to be protected from economic and noneconomic exploitation
9. Shelter and environment	Being able to be sheltered and to live in a safe and pleasant environment
10. Leisure activities	Being able to engage in leisure activities
11. Respect	Being able to be respected and treated with dignity
12. Religion and identity^†^	Being able to choose to live, or not to live, according to a religion and identity
13. Time autonomy^†^	Being able to exercise autonomy in allocating one’s time and undertake projects
14. Mobility^†^	Being able to be mobile

From Biggeri et al. (2006) copyright © 2006 United Nations Development Programme, reprinted by permission of Taylor & Francis Ltd, http://www.tandfonline.com on behalf of United Nations Development Programme

† Capability importance varies by age

**Table 2:** Two approaches for identifying different groups of children and young people

CYP group	Child development Biggeri et al. (2006)		Child capacity Matza et al. (2013)	
Group 1	Early childhood	(0-5 years old)	Proxy report only	(<5 years old)
Group 2	Childhood	(6-10 years old)	Possible child report	(5-7 years old)
Group 3	Early adolescence	(11-14 years old)	Improved child report	(8-11 years old)
Group 4	Adolescence	(15-17 years old)	Preferred child report	(12-18 years old)

CYP – children and young people

## Footnotes

1 Further research on the capability approach in this area is available from a bibliography developed by the Children and Youth Thematic Group of the Human Development and Capability Association, last updated in March 2019. Available online at: hd-ca.org/thematic_group/children
